# Management of duodenal stump fistula after gastrectomy for malignant disease: a systematic review of the literature

**DOI:** 10.1186/s12893-019-0520-x

**Published:** 2019-05-28

**Authors:** Maurizio Zizzo, Lara Ugoletti, Lorenzo Manzini, Carolina Castro Ruiz, Gabriela Elisa Nita, Magda Zanelli, Loredana De Marco, Giulia Besutti, Rocco Scalzone, Romano Sassatelli, Valerio Annessi, Antonio Manenti, Claudio Pedrazzoli

**Affiliations:** 1 Department of Oncology and Advanced Technologies, Surgical Oncology Unit, Arcispedale Santa Maria Nuova di Reggio Emilia, AUSL-IRCCS di Reggio Emilia, 42123 Reggio Emilia, Italy; 2 Clinical and Experimental Medicine PhD Program, University of Modena and Reggio Emilia, Modena, Italy; 3 General and Emergency Surgery Unit, Ospedale Civile di Guastalla, AUSL-IRCCS di Reggio Emilia, 42123 Reggio Emilia, Italy; 4 Department of Oncology and Advanced Technologies, Pathology Unit, Arcispedale Santa Maria Nuova di Reggio Emilia, AUSL-IRCCS di Reggio Emilia, 42123 Reggio Emilia, Italy; 5 Department of Imaging and Laboratory Medicine, Radiology Unit, Arcispedale Santa Maria Nuova di Reggio Emilia, AUSL-IRCCS di Reggio Emilia, 42123 Reggio Emilia, Italy; 6 Department of Oncology and Advanced Technologies, Gastrointestinal Endoscopy Unit, Arcispedale Santa Maria Nuova di Reggio Emilia, AUSL-IRCCS di Reggio Emilia, 42123 Reggio Emilia, Italy; 7 Department of General Surgery, Azienda Ospedaliero-Universitaria Policlinico, Del Pozzo Street 71, 41124 Modena, Italy

**Keywords:** Duodenal stump, Fistula, Gastric cancer, Gastrectomy, Management, Treatment

## Abstract

**Background:**

Duodenal stump fistula (DSF) remains one of the most serious complications following subtotal or total gastrectomy, as it endangers patient’s life. DSF is related to high mortality (16–20%) and morbidity (75%) rates. DSF-related morbidity always leads to longer hospitalization times due to medical and surgical complications such as wound infections, intra-abdominal abscesses, intra-abdominal bleeding, acute pancreatitis, acute cholecystitis, severe malnutrition, fluids and electrolytes disorders, diffuse peritonitis, and pneumonia. Our systematic review aimed at improving our understanding of such surgical complication, focusing on nonsurgical and surgical DSF management in patients undergoing gastric resection for gastric cancer.

**Methods:**

We performed a systematic literature review following the Preferred Reporting Items for Systematic Reviews and Meta-Analyzes (PRISMA) guidelines. PubMed/MEDLINE, EMBASE, Scopus, Cochrane Library and Web of Science databases were used to search all related literature.

**Results:**

The 20 included articles covered an approximately 40 years-study period (1979–2017), with a total 294 patient population. DSF diagnosis occurred between the fifth and tenth postoperative day. Main DSF-related complications were sepsis, abdominal abscess, wound infection, pneumonia, and intra-abdominal bleeding. DSF treatment was divided into four categories: conservative (101 cases), endoscopic (4 cases), percutaneous (82 cases), and surgical (157 cases). Length of hospitalization was 21–39 days, ranging from 1 to 1035 days. Healing time was 19–63 days, ranging from 1 to 1035 days. DSF-related mortality rate recorded 18.7%.

**Conclusions:**

DSF is a rare but potentially lethal complication after gastrectomy for gastric cancer. Early DSF diagnosis is crucial in reducing DSF-related morbidity and mortality. Conservative and/or endoscopic/percutaneous treatments is/are the first choice. However, if the patient clinical condition worsens, surgery becomes mandatory and duodenostomy appears to be the most effective surgical procedure.

## Background

Standard gastrectomy is the main surgical procedure performed with curative intent for gastric cancer [1]. It involves the resection of at least two-thirds of the stomach with a D2 lymph node dissection [1]. The reconstructions after total or distal gastrectomy imply the formation of a duodenal stump (with the exception of the Billroth I gastroduodenostomy) [1].

Duodenal stump fistula (DSF) remains one of the most serious complications after subtotal or total gastrectomy, as it puts patient’s life at risk [2]. DSF was defined as follows: presence of fluid through surgical abdominal drain or after radiological drainage with at least three times higher bilirubin or amylase concentration compared to normal serum value; or its leakage through the abdominal wall, regardless of its clinical impact, and confirmed by abdomen computed tomography (CT) scan and/or fistulography [2, 3]. The incidence of this complication varies between 1.6 and 5% [2, 4]. However, available scientific data are heterogeneous and clinical cases are not always comparable [5]. DSF is related to high mortality (16–20%) and morbidity (75%) rates, as a recent Italian multicenter study corfirmed [2]. Moreover, DSF-related morbidity always leads to longer hospitalization times, due to medical and surgical complications such as wound infections, intra-abdominal abscesses, intra-abdominal bleeding, acute pancreatitis, acute cholecystitis, severe malnutrition, fluids and electrolytes disorders, diffuse peritonitis, and pneumonia [6, 7].

Our work’s aim was to achieve deeper knowledge of this feared complication through an extensive systematic literature review, focusing on DSF nonsurgical and surgical management in patients undergoing gastric resection for gastric cancer.

## Methods

We performed a systematic literature review following the Preferred Reporting Items for Systematic Reviews and Meta-Analyzes (PRISMA) guidelines [8]. PubMed/MEDLINE, EMBASE, Scopus, Cochrane Library (Cochrane Database of Systematic Reviews, Cochrane Central Register of Controlled Trials-CENTRAL) and Web of Science (Science and Social Science Citation Index) databases were used to search all related literature, by combining the following non-MeSH / MeSH terms: ((duodenal stump fistula OR duodenal stump leakage OR duodenal stump leak) AND (management OR treatment) AND (gastric cancer OR gastric tumor OR gastric neoplasm OR stomach cancer OR stomach neoplasm)) OR (duodenal stump AND “Fistula”[Mesh] AND “Stomach Neoplasms”[Mesh]).

Our literature review was restricted to articles published over the past 30 years (January 1988–November 2018). Only English-written scientific papers were selected, including case reports, case series, case-control studies, cohort studies, controlled clinical trials, and randomized clinical trials. Prior systematic reviews and meta-analyses were excluded. The selected articles included adult patients treated for DSF after total or subtotal gastrectomy for gastric cancer. For those patients, DSF treatment methods and DSF treatment-related outcome (DSF resolution vs no resolution/mortality, and/or healing time) had to be reported, while articles not reporting DSF treatment methods and/or post-management outcome were excluded. In addition, references of relevant articles were searched, in order to identify cases of interest.

Two independent reviewers (MZ and LU) selected and identified papers based on title, abstracts, keywords, and full-text, then collecting following information from the selected papers: author’s surname and year of publication, study period, study type, DSF patient, timing of DSF diagnosis, neoadjuvant chemotherapy administration, stage of gastric cancer, DSF output, DSF-related complications, therapeutic strategy (conservative, endoscopic, percutaneous, surgical), clinical outcome, length of hospitalization, healing time, DSF-related mortality rate. Eventually, all collected results were reviewed by a third independent reviewer (AM).

## Results

**Fig. 1:**
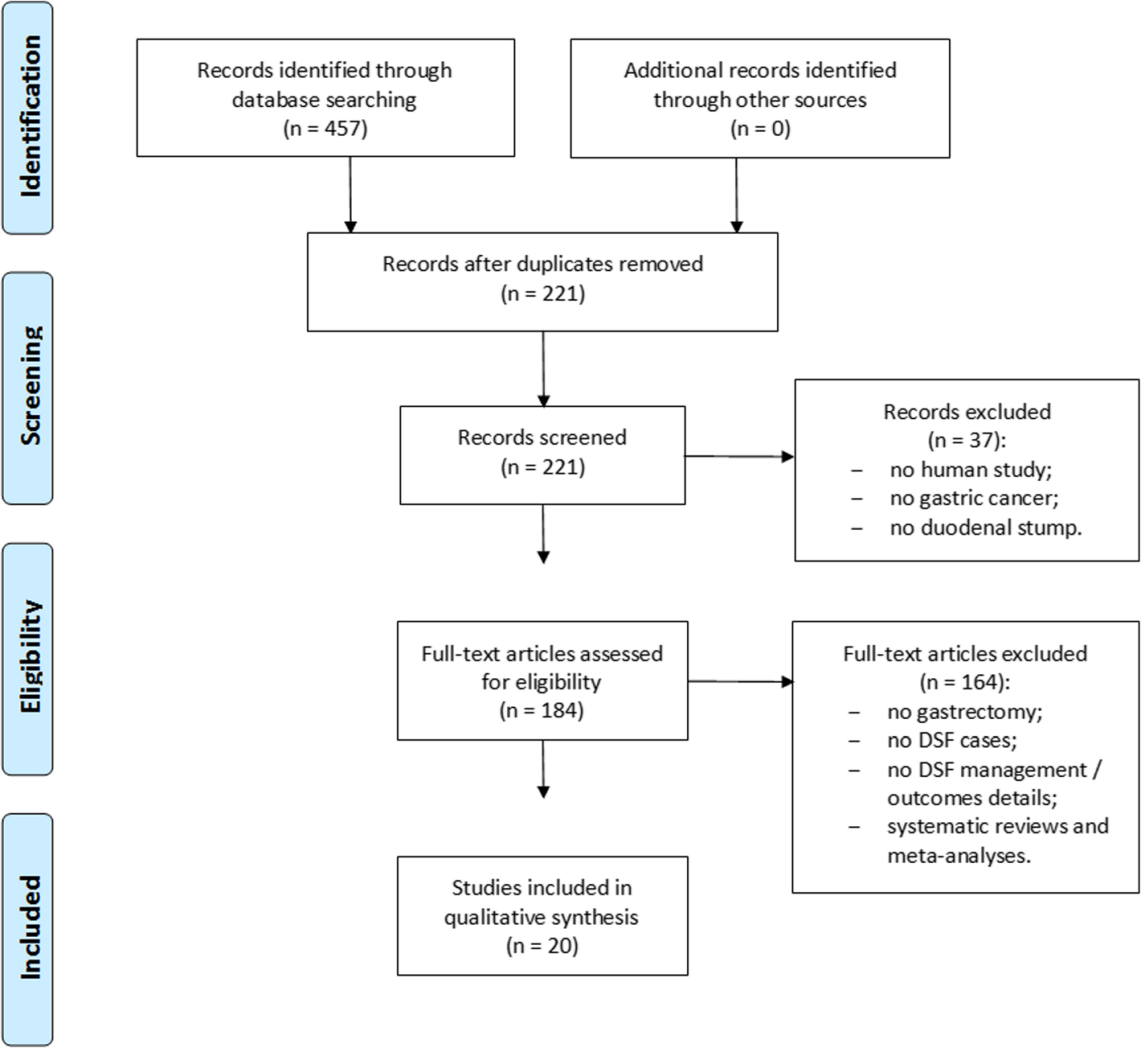
PRISMA flow chart of literature search

Final literature search, performed in November 2018, identified 457 potential items of interest (Fig. 1). After removing duplicate publications (236), 221 records were further analyzed. Thirty-seven out of which were excluded as not relevant, while 184 full-text articles were assessed for eligibility. After removing full-text articles not complying with inclusion criteria, 20 articles were included into qualitative synthesis [2–7, 9–22]. No item was included on the basis of other sources (eg. References lists). The included articles were case reports (5), single-center retrospective studies (13), and multicenter retrospective studies (2).

### General characteristics

General characteristics of the analyzed populations are shown by Table 1. Twenty included articles covered an approximately 40 years-study period (1979–2017), with a total 294 patient population [2–7, 9–22]. DSF diagnosis occurred between the fifth and tenth postoperative day (median days) [2–7, 9–22]. In accordance with output volume of gastrointestinal fistulas which are classified as low (<200 ml/24 h), moderate (200–500 ml/24 h), high (>500 ml/24 h), DSF output was mentioned exclusively by 5 papers [3–5, 9, 16]. Cozzaglio et al reported it in all 3 of their manuscripts, with a 290 to 500 (40–2200) median ml [4, 5, 16]. Main DSF-related complications were sepsis, abdominal abscess, wound infection, pneumonia, and intra-abdominal bleeding [2–7, 9–22]. DSF treatment was divided into four categories: conservative (101 cases), endoscopic (4 cases), percutaneous (82 cases), and surgical (157 cases) [2–7, 9–22]. Management and outcome data of the analyzed populations are shown by Table 2. Length of hospitalization was 21–39 days (median days), ranging from 1 to 1035 days [2–7, 9–22]. Healing time was 19–63 days (median days), ranging from 1 to 1035 days [2–7, 9–22]. DSF-related mortality rate was 18.7% (55/294 cases) [2–7, 9–22].

**Table 1:** Demographic and clinical data of reported cases of DSF after gastrectomy for malignant disease

Author/Year	Study period	Study type	Patients with DSF, n	Neoadjuvant chemotherapy, n	AJCC TNM stage	DSF diagnosis after operation, median days (range)	DSF output, median ml/day (range)	DSF-related complications
Garden et al./1988 [9]	1979–1985	RS	5	NA	NA	NA	1130 (120–2500)^a^	NA
Bloch et al./1989 [10]	1989	Case	1	0	I 1	NA	NA	NA
Kyzer et al./1997 [11]	1991–1994	RS	2	NA	NA	NA	NA	Sepsis (50%)
Wong et al./2000 [12]	1993–1997	RS	1	NA	NA	NA	210	Abdominal abscess
Oh et al./2009 [13]	1987–2004	RS	5	NA	NA	NA	NA	Sepsis (100%)
Lee et al./2009 [14]	2009	Case	1	0	I 1	7	NA	Abdominal abscess
Cozzaglio et al./2010 [5]	1991–2006	RS	68	2	NA	7 (0–22)	290 (40–2200)	Abdominal abscess (38%), Wound infection (28%), Sepsis (26%), Central line infection (15%), Pneumonia (13%), Acute renal failure (10%), Colonic fistula (7%), Pancreatic fistula (6%), Acute pancreatitis (6%), Intraabdominal bleeding (6%), Abdominal wall necrosis (4%), Pulmonary embolism (3%), Jejunal fistula (3%), Roux-en-Y syndrome (3%), Esophagojejunal fistula (3%), Heart failure (3%), Others (11%)
Hur et al./2010 [15]	2005–2007	RS	4	NA	NA	NA	NA	Abdominal abscess (100%)
Cozzaglio et al./2011 [16]	2005–2010	RS	6	NA	NA	6 (2–22)	500 (300–1000)	Sepsis (50%)
Curcio et al./2012 [17]	2012	Case	1	0	I 1	NA	NA	Abdominal abscess
Oh et al./2013 [18]	2005–2011	RS	10	NA	NA	10 (6–20)	NA	Abdominal fluids or abcess (100%)
Blouhos et al./2013 [19]	2013	Case	1	NA	IV 1	1	NA	Sepsis, Intra-abdominal bleeding
Vasiliadis et al./2014 [20]	2014	Case	1	NA	III 1	NA	NA	Dehydration and electrolyte disorders
Kim et al./2014 [21]	2002–2012	RS	13	NA	I 8, II 4, III 1	5 (1–12)	NA	NA
Orsenigo et al./2014 [3]	1987–2012	RS	32	0	I 7, II 9, III 9, IV 7	6.6 ± 4.7^a^	246 ± 266^a^	Sepsis (75%), Abdominal abscess (69%), Pneumonia (34%), Surgical site infection (28%), Intra-abdominal bleeding (22%), Acute renal failure (16%), Colonic fistula (12%), Gastro-jejunal anastomosis leakage (6%), Central line infection (6%), Pneumothorax (6%)
Cornejo et al./2016 [22]	1997–2014	RS	13	NA	I 4, II 5, III 2, IV 2	5 (3–7)^a^	NA	Sepsis (23%), Major Hematemesis (15%), Evisceration (8%), Abdominal abscess (8%)
Cozzaglio et al./2016 [4]	1990–2011	RS	75	3	NA	6 (2–11)	300 (100–750)	Abdominal abscess (70.7%); Sepsis (61.3%); Pneumonia (44%); Surgical site infection (38.7%); Digestive fistulas (29.3%); 2Acute renal failure (28%); Intra-abdominal bleeding (24%); Central line infection (17.3%); Acute pancreatitis (9.3%); Abdominal wall necrosis (8%); Others (21.3%)
Ali et al./2016 [7]	2010–2014	RS	24	NA	I 8, II 9, III 6, IV 1	8.5 (1–20)	NA	Pneumonia (20.8%), Sepsis (8.3%), Intra-abdominal bleeding (8.3%), Surgical site infection (8.3%), Deep vein thrombosis (8.3%)
Paik et al./2016 [6]	2008–2013	RS	16	NA	NA	6.5 (1–13)	NA	NA
Ramos et al./2018 [2]	2009–2017	RS	15	2	I/II 9, III/IV 6	9 (1–75)	NA	NA

^a^mean, *NA* not available

**Table 2:** Management and outcome data of reported cases of DSF after gastrectomy for malignant disease

Author/Year	Management						Outcomes	Hospital stay,	Healing time,	DSF-related
								median days (range)	median days (range)	mortality rate (%)
	Conservative	Endoscopic	Percutaneous			Surgical				
			Transhepatic biliary diversion	Abscess/Abdominal drainage	Duodenostomy					
Garden et al./1988 [9]	5	0	0	5	0	0	Conservative: 5/5 solved	NA	35 (17–71)^a^	0% (0/5)
							Endoscopic: −/−			
							Percutaneous: 5/5 solved			
							Surgical: −/−			
Bloch et al./1989 [10]	0	1	0	0	0	0	Conservative: −/−	NA	35	0% (0/1)
							Endoscopic: 1/1 solved			
							Percutaneous: −/−			
							Surgical: −/−			
Kyzer et al./1997 [11]	1	0	0	0	0	1	Conservative: 1/1 solved	NA	NA	50% (1/2)
							Endoscopic: −/−			
							Percutaneous: −/−			
							Surgical: 0/1 solved			
Wong et al./2000 [12]	0	1	0	0	0	0	Conservative: −/−	NA	2	0% (0/1)
							Endoscopic: 1/1 solved			
							Percutaneous: −/−			
							Surgical: −/−			
Oh et al./2009 [13]	0	0	0	0	0	5	Conservative: −/−	NA	18 (10–28)^a^	0% (0/5)
							Endoscopic: −/−			
							Percutaneous: −/−			
							Surgical: 5/5 solved			
Lee et al./2009 [14]	0	1	0	0	0	0	Conservative: −/−	34	17	0% (0/1)
							Endoscopic: 1/1 solved			
							Percutaneous: −/−			
							Surgical: −/−			
Cozzaglio et al./2010 [5]	51	0	4	15	2	27	Conservative: NA/NA	21 (7–65) without complications; 31 (1–1035) with complications	19 (1–1035)	16.2% (11/68)
							Endoscopic: −/−			
							Percutaneous: NA/NA			
							Surgical: NA/NA			
Hur et al./2010 [15]	0	0	0	0	4	1	Conservative: −/−	48 (30–158)	21 (12–44)	0% (0/4)
							Endoscopic: −/−			
							Percutaneous: 3/4 solved			
							Surgical: 1/1 solved			
Cozzaglio et al./2011 [16]	6	0	6	5	0	0	Conservative: 0/6 solved	63 (40–167)	63 (40–621)	50% (3/6)
							Endoscopic: −/−			
							Percutaneous: 0/5 PAD; 3/6 PTBD-OD solved			
							Surgical: −/−			
Curcio et al./2012 [17]	0	1	1	1	0	0	Conservative: −/−	NA	60	0% (0/1)
							Endoscopic: 1/1 solved			
							Percutaneous: 0/2 solved			
							Surgical: −/−			
Oh et al./2013 [18]	0	0	0	0	10	0	Conservative: −/−	32 (18–57)	51 (23–89)	0% (0/10)
							Endoscopic: −/−			
							Percutaneous: 10/10 solved			
							Surgical: −/−			
Blouhos et al./2013 [19]	0	0	0	0	0	1	Conservative: −/−	36	45	0% (0/1)
							Endoscopic: −/−			
							Percutaneous: −/−			
							Surgical: 1/1 solved			
Vasiliadis et al./2014 [20]	0	0	0	0	0	1	Conservative: −/−	17	15	0% (0/1)
							Endoscopic: −/−			
							Percutaneous: −/−			
							Surgical: 1/1 solved			
Kim et al./2014 [21]	3	0	0	0	0	10	Conservative: 3/3 solved	26 (12–140)	11.7 (8–18) conservative group, 57.3 (14–134) surgical group^a^	15.4% (2/13)
							Endoscopic: −/−			
							Percutaneous: −/−			
							Surgical: 8/10 solved			
Orsenigo et al./2014 [3]	11	0	3	5	0	13	Conservative: 11/11 solved	NA	31.2 ± 19.7 (conservative, PTBD and drainage); 45.2 ± 57.4 (surgical)^a^	9.4% (3/32)
							Endoscopic: −/−			
							Percutaneous: 8/8 solved			
							Surgical: 10/13 solved			
Cornejo et al./2016 [22]	5	0	0	0	0	8	Conservative: 5/5 solved	39.5 (26–65) conservative; 34.3 (13–84) surgical^a^	NA	46.2% (6/13)
							Endoscopic: −/−			
							Percutaneous: −/−			
							Surgical: 2/8 solved			
Cozzaglio et al./2016 [4]	0	0	0	0	0	75	Conservative: −/−	39 (22–68) solved; 32 (18–41) overall	28.5 (18–60) 1 surgical operation; 63 (50–82) > 1 surgical operation	28% (21/75)
							Endoscopic: −/−			
							Percutaneous: −/−			
							Surgical: 54/75 solved			
Ali et al./2016 [7]	5	0	0	11	3	5	Conservative: 5/5 solved	22 (11–96)	NA	0% (0/24)
							Endoscopic: −/−			
							Percutaneous: 14/14 solved			
							Surgical: 5/5 solved			
Paik et al./2016 [6]	6	0	6	1	0	3	Conservative: 6/6 solved	27.5 (15–54) solved group	NA	12,5% (2/16)
							Endoscopic: −/−			
							Percutaneous: 6/7 solved			
							Surgical: 2/3 solved			
Ramos et al./2018 [2]	8	0	0	0	0	7	Conservative: 5/8 solved	NA	NA	40% (6/15)
							Endoscopic: −/−			
							Percutaneous: −/−			
							Surgical: 4/7 solved			

^a^mean, *NA* not available

### Conservative treatment

Conservative treatment was mentioned in 10 studies and performed on 101 patients [2, 3, 5–7, 9, 11, 16, 21, 22]. Eight studies defined it as the only performed approach, while in 2 studies it was associated to other therapeutic methods. In most cases, it was used as first therapeutic choice, particularly in nonseptic and hemodynamically stable patients. From available data, a 91% success rate was defined [2, 3, 5–7, 9, 11, 16, 21, 22]. Conservative approach included fasting, enteral nutrition and / or parenteral nutrition, octreotide or somatostatin, particularly in case of high daily DSF output, and antibiotic therapy [2, 3, 5–7, 9, 11, 16, 21, 22].

Just Garden et al mentioned characteristics and indications for nutritional support [9]. Enteral nutrition started when access to proximal jejunum was feasible, when enteral diet did not increase fistula losses or it was poorly tolerated [9]. Enteral nutrition formulation was chosen based on patient tolerance [9]. In general, authors administered polymeric diets at 50 ml/h rate and 25 ml/h daily increase up to a 2000–3000 Kcal / day; 11–17 mg of nitrogen per day satisfactory intake [9]. Elemental formulations or peptides were administered in case of intolerance to polymeric diets [9]. If enteral nutrition was not tolerated or feasible, total parenteral nutrition turned out as preferential nutritional support [9].

According to Cozzaglio et al, 33 patients were treated by maintaining oral nutrition: among them, only 1 death occurred, in comparison to 10 deaths recorded among 35 fasting patients [5].

Kim et al and Orsenigo et al were the only researchers to report a healing time just related to conservative treatment [3, 21]. It was 11.7 and 31.2 ± 19.7 mean days, respectively [3, 21]. However, Orsenigo et al considered both medical and percutaneous treatment as part of conservative approach [3].

On the contrary, just Cornejo et al reported a length of hospitalization exclusively related to conservative treatment alone, which recorded 39.5 (26–65) mean days [22].

### Endoscopic treatment

Endoscopic treatment was mentioned in just 4 case report studies [10, 12, 14, 17]. For 3 patients, it was the only therapeutic approach, while in the remaining case it was applied after 2 failed percutaneous attempts. Available data allowed to determine a 100% success rate [10, 12, 14, 17]. Bloch et al used a peculiar endoscopic-percutaneous approach [10]. They performed a catheterization under transabdominal endoscopy beginning at external orifice of drainage incision and following drainage tube path [10]. Conversely, Wong et al inspected the fistula tract through choledochoscope, closed the tract using gelatin sponge and fibrin glue after irrigation and drainage of abscess [12]. Lee et al closed the fistulous orifice by placing metal clips [14]. Eventually, Curcio et al performed a circumferential clip placement along periphery of the fistula [17]. Two endoloops were placed over the endoclips and near the base, to fully close fistula [17]. In addition, fibrin glue was injected into the submucosa to ensure complete fistula sealing [17].

According to above mentioned authors, healing times were 35 days, 2 days, 17 days, and 60 days, respectively [10, 12, 14, 17]. Lee et al reported a 34-day length of hospitalization [14].

### Percutaneous treatment

Percutaneous treatment was cited by 9 studies [3, 5–7, 9, 15–18]. It was further divided into three different approaches: percutaneous transhepatic biliary diversion, percutaneous abscess / abdominal drainage and percutaneous duodenostomy. These three procedures were adopted in 20, 43 and 19 cases, respectively. In almost all cases, percutaneous treatment was associated and followed by conservative treatment if it failed. Data analysis showed a 91% success rate [3, 5–7, 9, 15–18].

Garden et al reported a 35 median day healing time, Cozzaglio et al reported a 63 median day one, Oh et al reported a 51 median day one, and Orsenigo et al reported a 31.2 ± 19.7 mean day one [3, 9, 16, 18].

Length of hospitalization related to exclusive percutaneous treatment ranged from 32 to 63 median days [16, 18].

### Surgical treatment

Surgical treatment was reported by 13 studies and applied in just over half whole population analyzed (53%, 157/294) [2–7, 11, 13, 15, 19–22]. Peritoneal lavage and abdominal drainage were performed in all reoperations, often in association to other surgical procedures. Main surgical procedures performed were primary closure of the duodenal stump (84/157, 53.5%), tube duodenostomy (58/157, 36.9%), biliary tree procedures - cholecystectomy, intracystic or intracholedochal Kehr T-tube placement, cholecystojejunal anastomosis (18/157, 11.5%), re-stapling of the duodenal stump (7/157, 4.4%), laparostomy (3/157, 1.9%).

In almost all cases, surgical treatment was performed in the presence of sepsis and / or haemodynamic instability. Available data showed a 71.5% success rate [2–7, 11, 13, 15, 19–22].

Healing time was extremely variable, ranging from 18 to 57.3 mean days [13, 21]. It was 28.5 and 63 median days for patients undergoing one or more than one reoperation, respectively [4].

Cornejo et al reported a 34.3 mean day length of hospitalization related to just surgical treatment [22].

## Discussion

DSF following total or subtotal gastrectomy for gastric cancer represents a rare complication with a reported incidence of 1.6–5% [2]. Despite the relatively low incidence rate, mortality rate remains high (from 7 to 67%) with a reported spontaneous closure rate of 28–92% [2, 5, 6, 22].

DSF pathogenesis remains unknown [2]. Main risk factors may be devascularization of duodenal stump or its inadequate surgical closure, inflammation of duodenal wall, local hematoma, neoplastic involvement of resection line, incorrect abdominal drain placement, and postoperative distension of the duodenum due to distal obstruction [2, 23].

Clinical DSF presentation time is variable with a mean 10-day diagnosis time [2]. Low fistula output may delay diagnosis, making it difficult to define fistula occurrence time [2]. Therefore, possible late clinical presentation must be kept in mind.

Many risk factors are related to DSF occurrence [2]. These may be related to patient characteristics (advanced age, cirrhosis, diabetes, heart disease, bio-humoral nutritional status impairment - preoperative albumin < 35 g / L and/or preoperative lymphocyte count < 2000 / mm3, preoperative chronic anemia, presence of chronic ulcer or ectopic pancreas in the duodenal bulb, previous hepatobiliary surgery), primary gastric cancer-related conditions (gastric outlet obstruction before gastrectomy, pylorus cancer invasion) and intraoperative procedures (blood loss > 300 ml, absence of manual suture line reinforcement, excessive vascular or pancreatic dissection around the duodenal stump, direct thermal damage of the duodenal stump) [3–6, 22].

Some studies underlined the importance of suture line reinforcement in DSF prevention [2]. In a recent prospective phase II study, Kim et al highlighted DSF absence in 100 patients undergoing laparoscopic reinforcement suture (LARS) with barbed suture during laparoscopic gastrectomy for gastric cancer [24]. Other authors suggested application of coated sutures, fibrin glues or resorbable reinforcements [2]. In a retrospective study on 2034 patients undergoing gastrectomy for gastric cancer, Shao et al analyzed three different techniques of duodenal stump closure [25]. They concluded that purse-string suture gave better outcomes in DSF rate when compared to duodenal stump treated with linear cutting stapler plus seromuscular layer reinforcement suture or full-thickness suture plus seromuscular layer reinforcement suture [25]. Orsenigo et al reported absent manual suture line reinforcement as an independent prognostic factor for DSF occurrence [3]. However, suture line reinforcement is not always easily performed as it happens in distal gastric lesions invading the pylorus or duodenum, where extended ultrapiloric resections are needed, as Ramos et al suggested [2]. Prospective randomized studies might help us determine effectiveness of suture line reinforcement, which is difficult to be performed due to small sample size related to low DSF incidence.

For subtotal gastrectomy, Marincas et al recently suggested the use of an intraoperatively introduced duodenal decompression probe, with the aim of reducing DSF risk [23]. However, the results were unsatisfactory [23].

DSF treatment can be classified into nonsurgical (conservative, endoscopic, percutaneous) and surgical. Nonsurgical treatment includes adequate fistula drainage, infection source control, and patient nutritional support. It represents the cornerstone of DSF management [2–7]. Instead, surgical treatment should be reserved only to those cases when nonoperative management does not allow an adequate fistula drainage leading to secondary complications such as intra-abdominal bleeding, sepsis, other fistulas, and intestinal obstruction [2–7, 22].

Scientific literature well describes impact of parenteral and enteral nutrition in preventing major complications after upper gastrointestinal, hepatobiliary, and pancreatic surgery [2]. Therefore, aggressive parenteral and / or enteral nutritional therapy, can significantly reduce DSF risk in addition to promoting its repair [2–5]. Analysis of published papers allowed to collect neither detailed data on DSF patient nutritional status nor indications and characteristics of nutritional support, except for what Garden et al reported [9]. However, malnutrition represents a key issue in patients with gastrointestinal fistulas, as it is closely associated to site and fistula output and it represents a major concern in patients affected by upper gastrointestinal fistulas such DSF [26]. A previous study identified a 53% malnutrition rate in patients with gastric or duodenal fistulas [26]. An “optimal nutritional support”, defined as a < 3000 Kcal or more per day and a positive nitrogen balance through a combination of oral, enteral and parenteral nutrition, was recommended in patients with gastrointestinal fistulas [26].

Patients with low-output fistulas should receive basal energy requirement and 1–1.5 g of protein / kg of body weight / day, with a minimum 30% caloric intake provided as lipids [26]. On the contrary, patients with high-output fistulas should receive 1.5–2 equivalent of their basal energy expenditure plus 1.5–2.5 g protein / kg body weight / day [26].

As patients often fail to achieve caloric support goals through enteral route for several days after starting feeding, immediate introduction of parenteral and enteral supports is strongly recommended for those patients, with the aim of interrupting parenteral support when enteral nutritional goals are met [26–29].

Equally important is that broad-spectrum antibiotics are administered and hydroelectrolytic and acid-base disorders corrected [2]. Effectiveness of treatment with somatostatin analogues was largely debated, although many authors suggested the administration of somatostatin analogues based on their potential efficacy in reducing intestinal secretion [3, 5]. On the contrary, the role of oral diet still appears uncertain, although it seems to be better than fasting, excluding patients with diffuse peritonitis and / or ileus [5].

Placement of abdominal drains in surgery for gastric cancer is under discussion [2]. It did not prevent DSF formation, although it could allow early DSF diagnosis thus avoiding other invasive diagnostic / therapeutic procedures [2]. Patients without abdominal drains or presenting DSF after their removal may be treated by a percutaneous approach: fluoroscopy, computed tomography, or ultrasound-guide drainage with pigtail catheter placement; transhepatic biliary drainage; fistula obliteration by cyanoacrylate or prolamine; occlusive balloon or Foley catheter placement [5, 15, 16, 18].

Biliary diversion with choledocostomy or percutaneous transhepatic biliary drainage and occlusive balloon were useful procedures in high-output fistulas described by literature [2]. Cozzaglio et al reported effectiveness of percutaneous transhepatic biliary drainage and occlusive balloon with from 500 to 100 ml / day reduced output in 6 patients [16]. However, complete resolution of DSF was achieved in half treated cases [16].

Conservative and / or endoscopic and / or percutaneous approach is / are considered first choice for DSF treatment and should be extended for at least 4–6 weeks, unless patient’s clinical situation worsens, thus requiring prompt surgery [2, 4, 5].

Surgery aims at draining multiple localized abscesses or treating a diffuse peritonitis (from severe abdominal sepsis or active bleeding) [2]. However, authors recommended to avoid surgery on fistulas occurring between 10 days and 6 weeks of initial gastric surgery [5]. During surgical reintervention, DSF can be managed / closed in different ways: washing of peritoneal cavity and abdominal drainage; closure of fistula (simple suture or re-stapling); biological glue; repair with rectus abdominis muscle flap; Roux-en-Y duodenojejunostomy; biliogastric diversion; laparostomy [2, 4]. However, effectiveness of these procedures is limited and it includes high risk of duodenal stump re-leakage due to postoperative edema and inflammation [4]. Therefore, treating acute setting with a duodenostomy would be more appropriate [4]. Ali et al suggested duodenostomy in order to avoid complex surgical interventions, concomitant increased morbidity and longer hospitalization, allowing future surgery where possibility for transfer exists or subspeciality expertise might be required [7]. Following duodenostomy, leakage site might close spontaneously within 2–6 weeks [18]. Other authors suggested pancreatoduodenectomy [4]. In addition to any surgical procedure for DSF, some authors recommended a prophylactic cholecystectomy, due to high risk of acute cholecystitis [5]. However, need for such additional procedure has not been confirmed yet.

An analysis of cases reported by literature did not allow to identify the most appropriate surgical strategy, probably due to high number of performed surgical procedures and low number of events [4]. However, patient outcome would seem better if peritoneal lavage and abdominal drainage were associated to surgical or percutaneous procedure on the biliary tree [4].

Despite improvements in nonsurgical diagnostic and therapeutic procedures, and surgical techniques, DSF-related mortality rate remains high, particularly during the first weeks following onset [5]. In small series, literature declared DSF and old age as independent factors associated to risk of surgical death (Clavien V) [2]. On the contrary, Cozzaglio et al found that DSF alone did not lead to patient’s death [5]. Development of new complications represented the real issue [5]. Moreover, the risk of death appeared to be closely related to the number of arising complications [5]. Therefore, best effort in preventing and treating septic complications is mandatory [5].

Some authors discussed the impact of laparoscopy on the risk of DSF development. Minimally-invasive surgery, laparoscopic gastrectomy in particular, is gaining increasing popularity in gastric cancer management [30]. Overall, relevant literature mainly stem from East Asia, while Western countries rarely performed randomized studies [30]. Currently, as early gastric cancer is concerned, in particular when it is located in distal stomach, different randomized trials proved laparoscopic gastrectomy superiority/noninferiority, in particular in reducing surgical trauma and enhancing postoperative recovery, with no compromise on surgical safety and oncologic efficacy [30]. Conversely, in advanced gastric cancer, multicenter large-scale randomized evidence endorsed laparoscopic gastrectomy safety and feasibility by experienced hands, while long-term survival outcomes, whose clarification requires support by several ongoing trials, remain pivotal in determining whether a wider applicability can be accepted [30, 31]. Cozzaglio et al estimated a 5 times higher risk in laparoscopic gastrectomies [4]. However, risk would seem related to specific learning curve, as suggested by other authors [3]. Another possible explanation could be nonroutine execution of suture line reinforcement in laparoscopic approach, although such assumption was not confirmed by Cozzaglio et al’s large multicenter study [4].

### Limitations

Our systematic review presents several limitations: i) reported events were mainly case reports or small retrospective series; ii) populations under analysis presented heterogeneity; iii) many relevant data were not described by the authors in detail, as reported in Tables 1 and 2; iv) number of reported procedures was higher than number of patients, given frequent association of different therapeutic approaches; therefore, some patients were simultaneously taken into consideration in different groups; v) data on timing of DSF diagnosis, healing time and length of hospitalization were reported in median days or mean days; therefore, direct confrontation of results appeared difficult.

## Conclusions

DSF represents a rare but potentially lethal complication after gastrectomy for gastric cancer. Early DSF diagnosis is crucial in minimizing DSF-related morbidity and mortality. However, early diagnosis is often difficult, because of clinical manifestations that only include moderate and nonspecific symptoms and signs. Conservative and / or endoscopic / percutaneous treatment is / are the first choice. In worsening of patient clinical condition, surgery becomes mandatory and duodenostomy appears to be the most effective surgical procedure.

## Data Availability

All data and materials are contained within the manuscript.

## Abbreviations

CT: Computed tomography
DSF: Duodenal stump fistula
LARS: Laparoscopic reinforcement suture
PRISMA: Preferred Reporting Items for Systematic Reviews and Meta-Analyzes
