# Correction to: Management of duodenal stump fistula after gastrectomy for malignant disease: a systematic review of the literature

**DOI:** 10.1186/s12893-019-0626-1

**Published:** 2019-10-24

**Authors:** Maurizio Zizzo, Lara Ugoletti, Lorenzo Manzini, Carolina Castro Ruiz, Gabriela Elisa Nita, Magda Zanelli, Loredana De Marco, Giulia Besutti, Rocco Scalzone, Romano Sassatelli, Valerio Annessi, Antonio Manenti, Claudio Pedrazzoli

**Affiliations:** 1 Department of Oncology and Advanced Technologies, Surgical Oncology Unit, Arcispedale Santa Maria Nuova di Reggio Emilia, Azienda Unità Sanitaria Locale – IRCCS di Reggio Emilia, 42123 Reggio Emilia, Italy; 2 Clinical and Experimental Medicine PhD Program, University of Modena and Reggio Emilia, Modena, Italy; 3 General and Emergency Surgery Unit, Ospedale Civile di Guastalla, Azienda Unità Sanitaria Locale – IRCCS di Reggio Emilia, 42123 Reggio Emilia, Italy; 4 Department of Oncology and Advanced Technologies, Pathology Unit, Arcispedale Santa Maria Nuova di Reggio Emilia, Azienda Unità Sanitaria Locale – IRCCS di Reggio Emilia, 42123 Reggio Emilia, Italy; 5 Department of Imaging and Laboratory Medicine, Radiology Unit, Arcispedale Santa Maria Nuova di Reggio Emilia, Azienda Unità Sanitaria Locale – IRCCS di Reggio Emilia, 42123 Reggio Emilia, Italy; 6 Department of Oncology and Advanced Technologies, Gastrointestinal Endoscopy Unit, Arcispedale Santa Maria Nuova di Reggio Emilia, Azienda Unità Sanitaria Locale – IRCCS di Reggio Emilia, 42123 Reggio Emilia, Italy; 7 Department of General Surgery, Azienda Ospedaliero-Universitaria Policlinico, Del Pozzo Street 71, 41124 Modena, Italy

**Correction to: BMC Surg (2019) 19:55**

**https://doi.org/10.1186/s12893-019-0520-x**

Following publication of the original article [1], the authors have notified us that due to administrative reasons they would like to modify the first affiliation from:

- AUSL-IRCCS di Reggio Emilia

To:

- Azienda Unità Sanitaria Locale – IRCCS di Reggio Emilia
